# Synthesis and Electrochemical Property of LiMn_2_O_4_ Porous Hollow Nanofiber as Cathode for Lithium-Ion Batteries

**DOI:** 10.1186/s11671-017-1879-1

**Published:** 2017-02-10

**Authors:** Lianfeng Duan, Xueyu Zhang, Kaiqiang Yue, Yue Wu, Jian Zhuang, Wei Lü

**Affiliations:** 1 Advanced Institute of Materials Science, Key Laboratory of Advanced Structural Materials, Ministry of Education, Changchun University of Technology, Changchun, 130012 China; 2 Department of Materials Science and Engineering, Changchun University of Technology, Changchun, 130012 China; 3 Key Laboratory of Bionics Engineering, Ministry of Education, Jilin University, Changchun, 130025 China

**Keywords:** LiMn_2_O_4_, Porous structure, Hollow nanofibers, Cathodes, Lithium-ion batteries

## Abstract

The LiMn_2_O_4_ hollow nanofibers with a porous structure have been synthesized by modified electrospinning techniques and subsequent thermal treatment. The precursors were electrospun directly onto the fluorine-doped tin oxide (FTO) glass. The heating rate and FTO as substrate play key roles on preparing porous hollow nanofiber. As cathode materials for lithium-ion batteries (LIBs), LiMn_2_O_4_ hollow nanofibers showed the high specific capacity of 125.9 mAh/g at 0.1 C and a stable cycling performance, 105.2 mAh/g after 400 cycles. This unique structure could relieve the structure expansion effectively and provide more reaction sites as well as shorten the diffusion path for Li^+^ for improving electrochemical performance for LIBs.

## Background

Lithium-ion battery (LIB) as energy storage devices is widely considered as the most promising rechargeable power sources because of high power density, no memory effect, long cycle life, high voltage, and low self-discharge, expecting to be utilized in portable electronic appliances and electric vehicles (EVs) [1, 2]. To improve the energy density and cycle performance of LIBs, much more efforts have been made to develop the new electrode materials, especially for cathode [3, 4]. The composition LiM_x_O_y_ (M = Co, Ni, V, Si, etc.) has attracted much attention as high-energy density cathode materials for lithium rechargeable batteries [5–7]. Among different kinds of cathode materials for lithium battery, LiMn_2_O_4_ is considered to be an attractive cathode material because of its low cost, abundant resources, low toxicity, and better safety, compared to other transition metal oxides [8, 9]. Wang et al. fabricated LiMn_2_O_4_ nanofiber with a specific capacity of 110 mAh/g at 0.5 C after 60 cycles [10]. In addition, Kanamura et al. prepared LiMn_2_O_4_ thin film by the PVP sol-gel method and the capacity fade was ca. 20% during 200 cycles [11]. However, due to the dissolution of Mn ion into the electrolyte and structural distortion, it suffers capacity fading on cycling resulting in a poor long-term cycle stability [12, 13].

The cathode materials with special structure and morphology could increase their specific capacity and cycling performance, aiming to overcome the mechanical strain arising from the huge volume variation and the particle agglomerations during the charge/discharge processes of lithium-ion battery, which results in the increased diffusion lengths and serious electrical disconnection [3, 14, 15]. The porous structure can buffer the large volume change and the agglomerations of electrode, which could be enhanced the cycling performance [16, 17]. So, one-dimensional (1D) nanofibers with porous structure have been demonstrated to be the new structure cathode for LIBs [18]. Because of its simple procedure and good controllability, the electrospinning technique is appealing in synthesizing nanofibers compared with other methods, such as chemical vapor deposition, pulsed laser deposition, hydrothermal process, sol-gel methods [19–21].

In this work, LiMn_2_O_4_ nanofiber with porous hollow structure and morphology has been successfully synthesized by modified electrospinning method. It is discussed that the effects of varied heat treatment factors in detail which explore the formation mechanism of nanofibers with a hollow structure. Furthermore, the electrochemical performances of the samples as cathode for LIBs were evaluated. It is worth that LiMn_2_O_4_ porous hollow nanofiber morphology delivers the higher electrochemical performance.

## Methods

### Preparation of LiMn_2_O_4_ Nanofibers

The LiMn_2_O_4_ nanofiber with porous hollow structure and morphology has been successfully synthesized by modified electrospinning method. All chemicals were of analytical grade and used as purchased without further purification. Firstly, the precursor solution was prepared by mixing 2 g of polyvinylpyrrolidone (PVP, Mw ≈ 1,300,000) in 20 ml ethanol under vigorous stirring for 4 h. 1.224 g Li(CH_3_COO) · 2H_2_O and 5.88 g Mn(CH_3_COO)_2_ · 4H_2_O were dissolved into the solution. And the acetic acid (1 ml) was then added to the above solution under continuous stirring for 2 h. After that, the precursor solution was transferred into a syringe with the metal syringe needle. The 20 kV high voltage was applied across the collector (aluminum foil or luorine-doped tin oxides (FTOs)) and metal needle, whose distance maintained at 20 cm. The precursor collected on collectors was dried at 60 °C for 1 h. LiMn_2_O_4_ cathode was obtained by calcining the precursor nanofibers in air under different heating rates and temperatures.

### Materials Characterization

The X-ray diffraction patterns (XRD) of fabricated powders were recorded by a Rigaku D/max 2500 pc X-ray diffractometer with Cu K radiation (=1.54156 Å) at a scan rate of 5 deg/s. The field emission scanning electron microscope (FESEM) was conducted on a JEOL JSM-6700F field emission scanning electron microscopy. Transmission electron microscopy (TEM) and high-resolution TEM (HRTEM) observations were performed by a JEOL 2100F. The thermogravimetric (TG) curves were obtained by a Perkin-Elmer TGA 7 thermogravimetric analyzer from room temperature to 800 °C at a heating rate of 10 °C/min. Brunauer-Emmett-Teller (BET) N_2_ adsorption-desorption surface area measurements were conducted on a volumetric sorption analyzer (NOVA 2000, Quantachrome).

### Electrochemical Property

The electrochemical experiments of LiMn_2_O_4_ nanofiber with porous hollow structure for the working electrode, which prepared by casting slurry containing 10% acetylene black, 10% polyvinylidene fluoride (PVDF), and 80% active material onto a aluminum foil, were performed via CR2025-type coin cells assembled in a dry argon-filled glove box. The test cell was assembled using working electrode and lithium foil which were separated by a Celgard 2400 microporous membrane. The electrolyte solution was prepared by dissolving 1 M LiPF_6_ in ethyl carbonate and diethyl carbonate (1:1 by volume). Galvanostatic charge-discharge cycling tests were performed using a LAND CT2001A multi-channel battery testing system in the voltage range between 3.0 and 4.4 V at room temperature.

## Results and Discussion

**Fig. 1:**
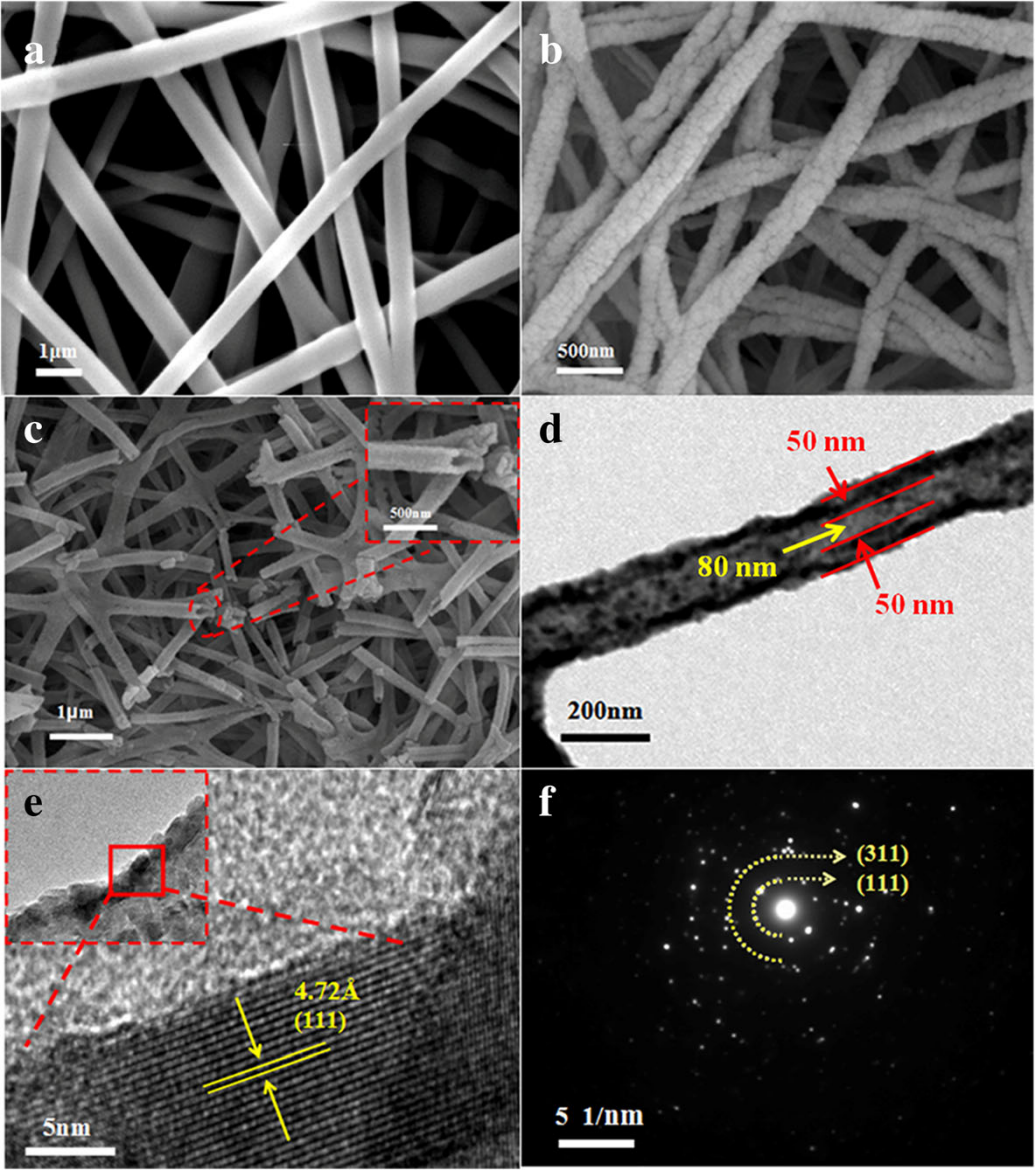
**a** FESEM image of electrospun LiMn_2_O_4_ nanofibers precursor. **b** LiMn_2_O_4_ nanofibers after being annealed at 700 °C for 5 h. **c** Nanofibers with a hollow structure (*Inset* magnified view of nanofibers indicating hollow structure), **d** TEM image, **e** HRTEM image, **f** SAED image of porous hollow nanofiber

Figure 1a, b shows the FESEM images of precursor nanofibers and the LiMn_2_O_4_ nanofibers annealed at 700 °C for 5 h with a heating rate of 5 °C/min, respectively. Figure 1a shows the morphology of precursor nanofiber, which exhibits smooth surface. The diameter of nanofiber is about 500 nm. After thermal treatment, the nanofiber has been formed with uniform diameter of about 200 nm and porous surface because of the burnout of organic components during the calcination process (as shown in Fig. 1b). In addition, the nanofibers form “network” structures, which facilitate the fast charge-discharge characteristics, because the structure could provide short lithium diffusion path and large surface area [10]. Figure 1c shows the hollow structure of nanofibers, which is clearly from the inset of Fig. 1c. The thickness of the tube wall is about 50 nm and the diameter of hollow structure is about 80 nm (Fig. 1d). From the HRTEM image (Fig. 1e), the lattice fringe spacing of the LiMn_2_O_4_ particles is almost in accordance with the d-spacing value (4.76 Å) of cubic LiMn_2_O_4_ in (111) plane. And the regular lattice fringes can also demonstrate the high crystallinity [22, 23]. Figure 1f shows the selected area electron diffraction (SAED) pattern; it indicates that the nanofibers are single crystalline and the corresponding miller indices are indexed accordingly [24].

**Fig. 2:**
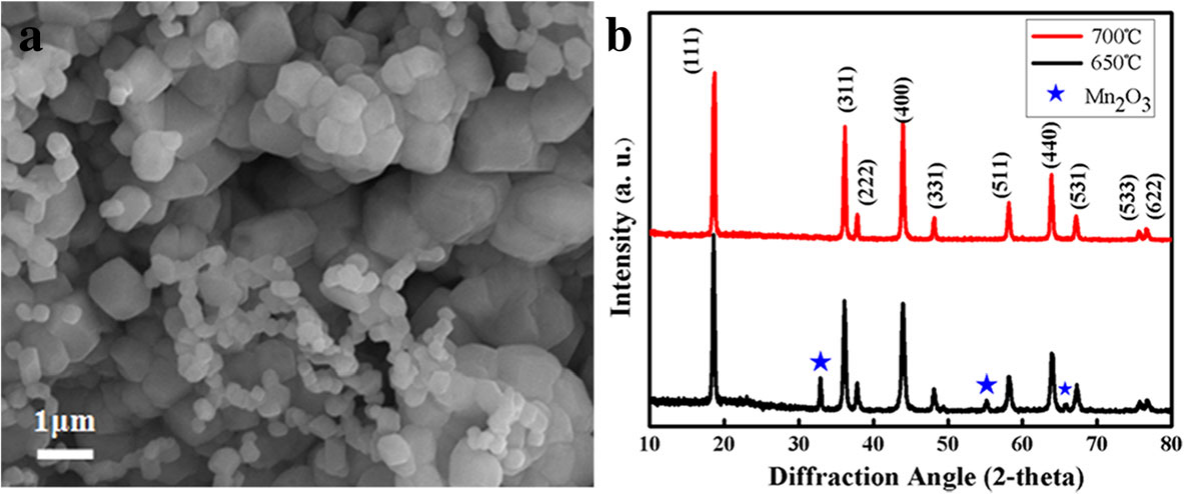
**a** FESEM images of samples calcined at 650 °C, **b** X-ray diffraction pattern of calcined LiMn_2_O_4_ nanofibers at 650 and 700 °C

**Fig. 3:**
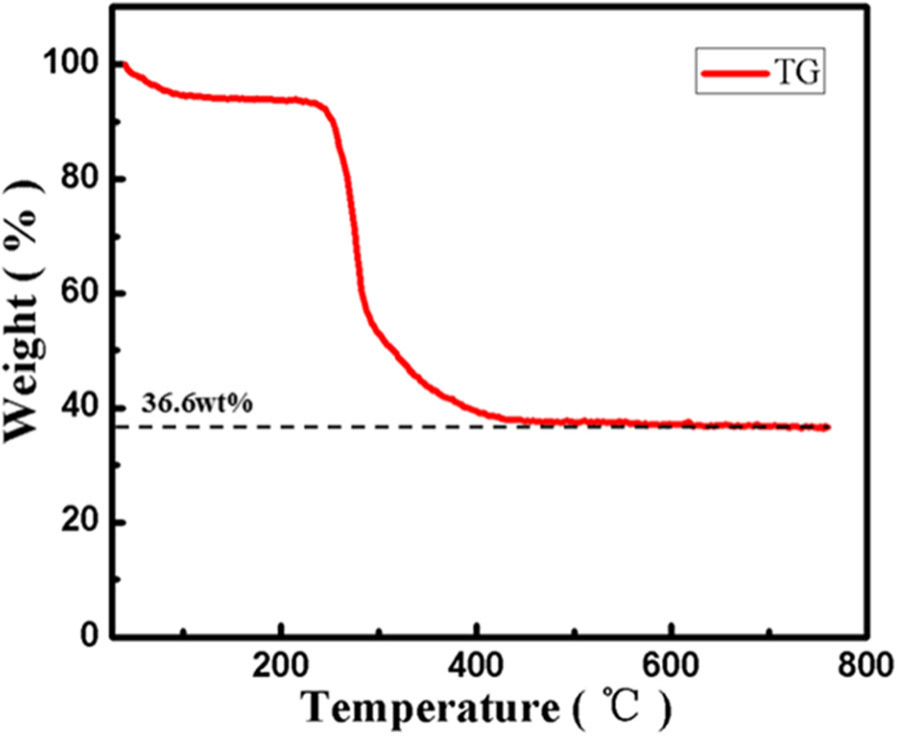
TG curve for as-spun LiMn_2_O_4_ nanofibers

Figure 2a is the FESEM of the LiMn_2_O_4_ nanoparticles calcined at 650 °C and 5 °C/min, the particles with heterogeneous size could be prepared, without nanofibers. Figure 2b is the X-ray diffraction pattern of the LiMn_2_O_4_ nanofibers calcined at different temperatures. As it can be seen from Fig. 2b, the diffraction peaks (calcined at 700 °C) can be indexed to LiMn_2_O_4_ phase (JCPDS 88-1749) with no other impurities. Meanwhile, the sharpness of the X-ray diffraction peaks confirms that the material should be crystallized LiMn_2_O_4_. However, some peaks which belong to Mn_2_O_3_ phase are also can be seen in the nanofibers obtained at 650 °C, which is evident that the presence of unavoidable impurity phase (Mn_2_O_3_) is can be seen when the as-spun LiMn_2_O_4_ nanofibers are treated at a temperature of 650 °C [25]. The calcination and the decomposition temperature of precursor were determined by thermogravimetric (TG) analysis. In Fig. 3, the first weight loss (0–130 °C) could be resulted from the evaporation of hydrate. Then, a noticeable weight loss (36.6 wt% of original weight) is observed between 250 and 450 °C because of the decomposition of PVP. So, the pre-heated temperature is at 500 °C for removing the PVP completely. From above, the LiMn_2_O_4_ nanofibers without any other impurities could be synthesized at 700 °C.

**Fig. 4:**
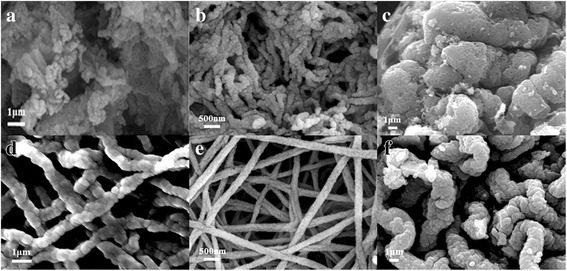
FESEM images of samples after being calcinated at different heating rates **a**, **d** 3 °C/min, **b**, **e** 5 °C/min, **c**, **f** 7 °C/min with Al foil (**a**, **b**, **c**) and FTO (**d**, **e**, **f**)

To understand the formation mechanism of the porous hollow nanofibers structure, the as-prepared samples were analyzed by FESEM at different heating rates (3, 5, and 7 °C/min) with different substrates such as Al foil (a, b, c) and FTO (d, e, f) in Fig. 4. In this figure, with increasing the heating rate from 3 °C/min to 5 °C/min, the size of nanoparticles and diameter of nanofibers are decreased, and then, they are increased from 5 °C/min to 7 °C/min. At the high-temperature heating (7 °C/min), the highly porous structure should be destroyed to create a dense morphology for use in Li-ion batteries. Therefore, the porous hollow wires in this process are ideal for obtaining highly crystalline nanomaterials to be used as the active material at 5 °C/min. First of all, at the slow heating rate was employed, the decomposition of polymer and the release of CO_2_ and H_2_O are slower, so the LiMn_2_O_4_ particles formed earlier have more time to grow. Following it, the size of nanoparticles is larger and the morphologies of the samples are bending and rough because of the quickly decomposition of polymer and growth of crystal. It is shown that the morphology of porous surface is affected by the higher release of CO_2_ and H_2_O with the higher heating rate. In addition, the porous hollow nanofibers could be more synthesis on FTO as substrate. The agglomeration of nanofibers is much more serious with Al foil as substrate in Fig. 4. Compared to Al foil, the FTO is a kind of semiconductors as transparent conducting oxide with higher resistivity, stable chemical performance, and strong acid and alkali resistance at room temperature. Just for it, at the same voltage for applying across the collector and metal needle, the jet velocity of the precursor solution is slower than that of the Al foil. The fiber morphology of the precursor collected on FTO could be kept more integrity and uniformity. The FTO could be used for substrate at the electrospinning process. So, we first prepared the LiMn_2_O_4_ porous hollow nanofibers by electrospinning it onto FTO glass directly to form porous electrode.

**Fig. 5:**
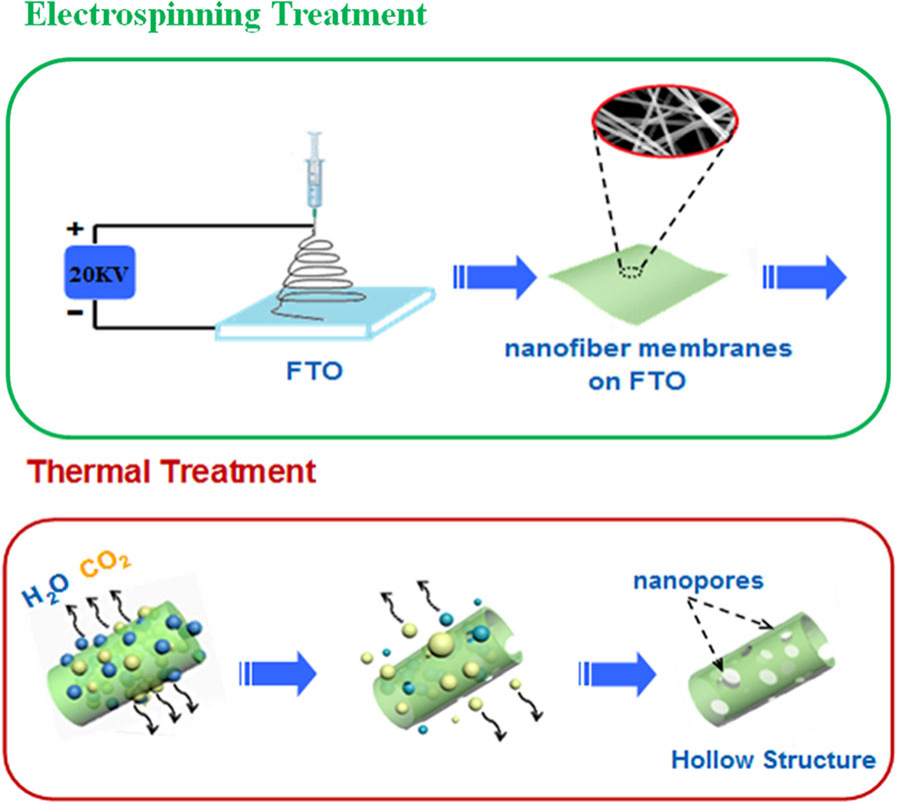
Schematic of the formation processes of the hollow nanofibers with porous structure

The formation of the hollow nanofibers with porous structure is introduced in Fig. 5. There are three stages for the formation process of unique structure: (1) The precursor nanofibers are prepared on the FTO by electrospinning. (2) Under thermal treatment, the polymer at the surface of precursor nanofibers decomposes into H_2_O and CO_2_ and releases, meanwhile the inner organic moieties together with metal ions present in the precursor move toward the outer wall under the effect of concentration difference [26]. In that case, the gas diffusion rate from the interspaces between the nanoparticles is slower than that of PVP decomposition, which makes the pressure inside of the composite fibers larger than that outside of the composite fibers, and the nanoparticles would move toward outside of the composite fibers to form hollow fibers. (3) With the temperature further increasing, the Li^+^ and Mn^2+^ aggregated react with each other in the air atmosphere resulting in the formation of LiMn_2_O_4_, which continues to grow and interconnects to form the final continuous tubular structure with porous and thin wall [27, 28]. The formation mechanism of hollow nanofibers and the key roles of FTO as substrate need to be further studied in the future. It is also the base of synthesizing the nanofiber composites as cathode for LIBs.

**Fig. 6:**
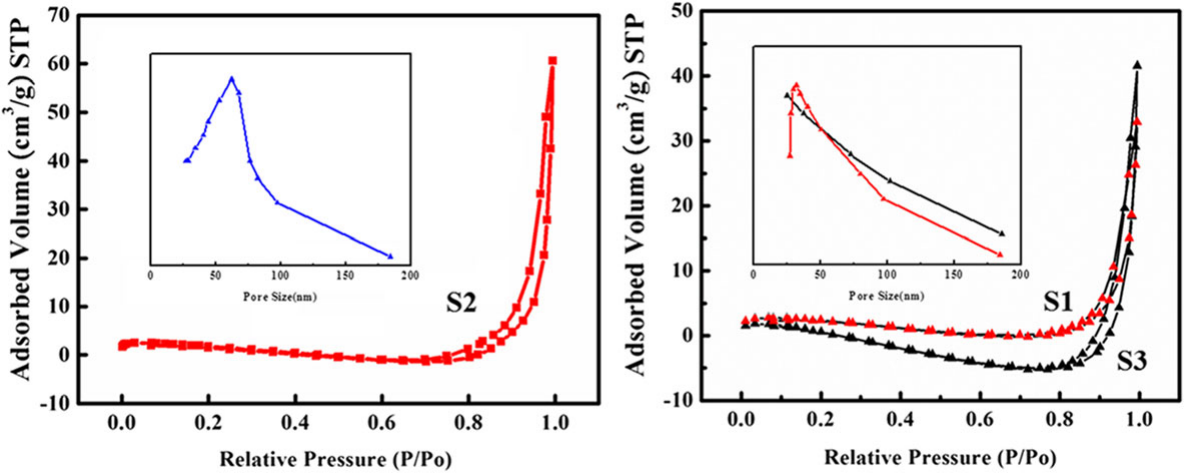
N_2_ adsorption-desorption isotherms for LiMn_2_O_4_ nanofibers S2 (a), S1 and S3 (b), with *insets* showing the BJH pore size distributions for the corresponding samples

N_2_ adsorption-desorption and the corresponding pore size distribution curve of S1, S2, and S3 LiMn_2_O_4_ nanofibers (heating rates are 3, 5, and 7 °C/min, respectively) are shown in Fig. 6. Figure 6a shows the large hysteresis loops between the N_2_ adsorption and desorption isotherms in the *P*/*P*_0_ ranging from 0.7 to 1. It confirms the broad pore size distribution toward larger mesopores (inset of Fig. 6) and the formation of mesopore textural porosity. The BET specific surface area of S2 is 84.3 m^2^/g, and the average pore size is around 60 nm, which are in good agreement with the TEM analysis. However, the BET specific surface areas and pore sizes of S1 and S3 are much smaller than S2. The larger mesopore derives from the gas diffusion decomposed from PVP, which drives the nanoparticles to transfer from interior to exterior of the composite fibers.

**Fig. 7:**
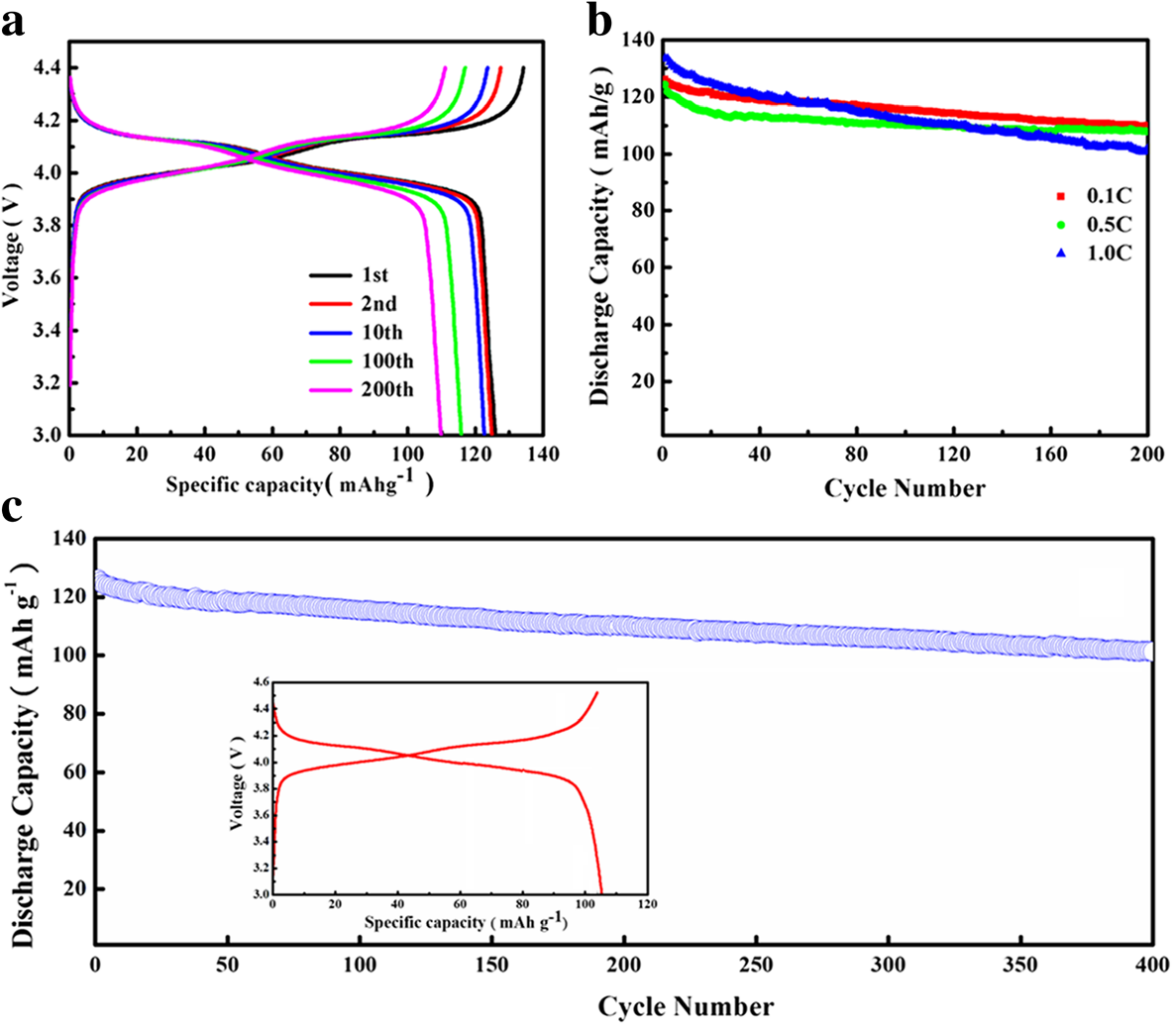
**a** Galvanostatic charge-discharge curves of S2 at the current density 0.1 C and **b** cycle performance of S2 at 0.1 C, 0.5 C, and 1.0 C, **c** cycling performance of S2 for 400 cycles at 0.1 C. *Inset* shows the charge-discharge curves of the 400th cycle

The electrochemical performances of the LiMn_2_O_4_ porous hollow nanofibers (S2) were evaluated by galvanostatic charge/discharge cycling in the voltage range of 3.0–4.4 V at 0.1 C (1 C = 148 mAh/g). In Fig. 7a, there are two distinct voltage plateaus at 4.1 and 4.0 V, respectively, corresponding to the two-step intercalation and deintercalation of Li^+^ into or out LiMn_2_O_4_ during the charge and discharge process. The two flat voltage plateaus are corresponding to the two-phase and one-phase reaction modes, respectively, which are the region equilibrium between LiMn_2_O_4_ and Li_0.5_Mn_2_O_4_ phases (LiMn_2_O_4_⇋Li_0.5_Mn_2_O_4_ + 0.5Li^+^+0.5e^−^) and the equilibrium between Li_0.5_Mn_2_O_4_ and γ-MnO_2_ (Li_0.5_Mn_2_O_4_⇋2MnO_2_ + 0.5Li^+^+0.5e^−^) [29]. The electrode exhibits a discharge capacity of 125.9 mAh/g and charge capacity of 132.8 mAh/g at 0.1 C for the first cycle. The well overlapping of charge-discharge curves and the mild capacity fading between 1 and 10 cycles indicate that there is less severe formation of SEI films and polarization effect occurs during the circulation [30]. After 100 and 200 cycles, it still retains a capacity of 117.2 and 114.9 mAh/g, which decreases slightly. Similar trend is observed at 0.5 C and 1 C, reaching a specific capacity of 115 and 103.8 mAh/g after 200 cycles, respectively (Fig. 7b). Figure 7c shows the exceedingly stable long-term cycling performance of the LiMn_2_O_4_ porous hollow nanofibers, which shows the capacity remains of 105.2 mAh/g at 0.1 C after 400 cycles. As it can be seen from Fig. 7c inset, there are also two distinct separated voltage plateaus. It is evidenced that the porous hollow nanofibers possess an outstanding structure and component stability during charge-discharge process.

**Fig. 8:**
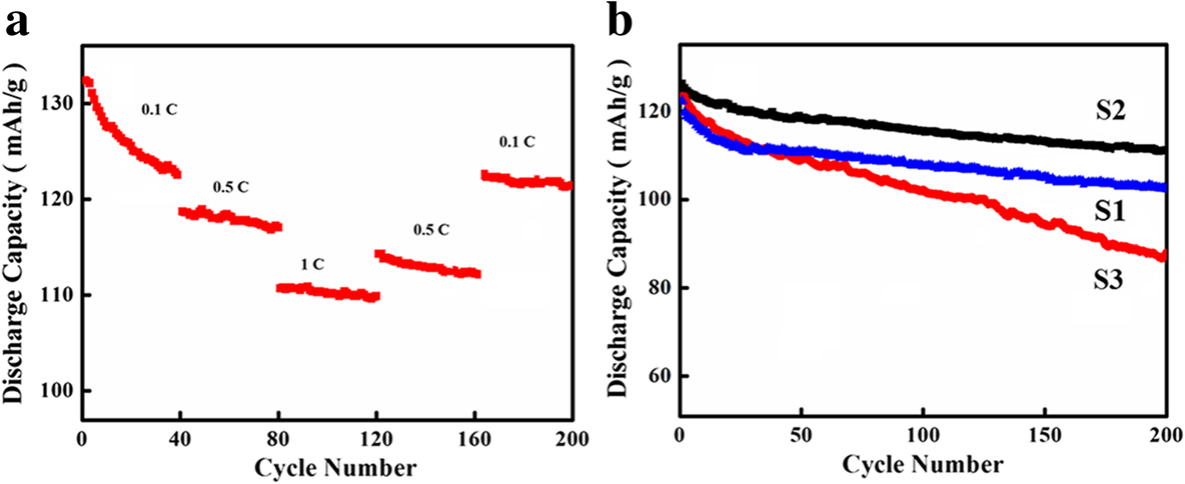
**a** Rate performance of S2. **b** Cycling performance of S1, S2, and S3 for 200 cycles at 0.1 C

Figure 8a presents the rate capability of the LiMn_2_O_4_ hollow nanofibers. The LiMn_2_O_4_ electrode shows a capacity loss from 122 mAh/g at 0.1 C to 102 mAh/g at 1 C, which is only 16.3% of an initial discharge capacity at 0.1 C. Furthermore, after 80 cycles (from the 81st cycle to the 161st cycle), the discharge capacity of LiMn_2_O_4_ could be recovered (123 mAh/g), when the charge-discharge rates reduce from 1 C to 0.1 C. Figure 8b shows the cycling performance of the S1, S2, and S3 with different morphologies at a current rate of 0.1 C. The S2 (Fig. 4e) electrode delivers an initial discharge capacity of 121 mAh/g. After 200 cycles, the capacity retention is 107 mAh/g. Compared to S1 and S2, S3 shows an initial discharge capacity of 123 mAh/g and retains 86 mAh/g after 200 cycles, and the capacity retention of 70% is much lower than the S2 electrode. It is worth noting that the LiMn_2_O_4_ porous hollow nanofiber electrode delivers the excellent rate capability, especially for showing stable electrochemical property at different current densities.

On the basis of the above results, the high reversible capacity, rate behavior, and excellent cycling life of the LiMn_2_O_4_ porous hollow nanofibers are due to the novel porous hollow structure. Firstly, the porous nanostructure can provide more reaction sites for the intercalation/deintercalation of Li^+^ and shorten the diffusion path for Li^+^, leading to the better rate capability [31, 32]. Secondly, the hollow structure with high crystallinity possesses a high structural stability. The manganese could not be dissolved easily from the spinel phase because of the robust structure. Thirdly, the hollow structure could effectively relieve the structural strain and volume change which can be much more helpful for its long-term circulation. All of them contribute to the high reversible capacity, rate capability, and good cycle performance of porous hollow LiMn_2_O_4_ nanofibers as the cathode for LIBs.

## Conclusions

In summary, the LiMn_2_O_4_ hollow nanofibers with a porous structure have been synthesized by modified electrospinning techniques on the FTO substrate. And the precursor was calcined in air at 700 °C. As the cathode materials for LIBs, the LiMn_2_O_4_ with special morphology exhibits good cycle life and high capacity, which delivers a high specific capacity of 125.9 mAh/g and a stable cycling performance of 105.2 mAh/g after 400 cycles, at 0.1 C. It is certificated that the porous and hollow structure improves the utilization of the active mass and dual conduction of Li^+^ and electrons and effectively relieves the structural strain and volume change, which could enhance the cycling performance and rate stability for advanced energy storage devices.

## Abbreviations

LIB: Lithium-ion battery
EV: Electric vehicles
FTO: Fluorine-doped tin oxide
1D: One-dimensional
PVP: Polyvinylpyrrolidone
XRD: X-ray diffraction patterns
FESEM: Field emission scanning electron microscopy
TEM: Transmission electron microscopy
HRTEM: High-resolution transmission electron microscopy
BET: Brunauer-Emmett-Teller
TG: Thermogravimetric
PVDF: Polyvinylidene fluoride
SAED: Selected area electron diffraction
